# Afatinib versus placebo as adjuvant therapy after chemoradiation in a double-blind, phase III study (LUX-Head & Neck 2) in patients with primary unresected, clinically intermediate-to-high-risk head and neck cancer: study protocol for a randomized controlled trial

**DOI:** 10.1186/1745-6215-15-469

**Published:** 2014-11-29

**Authors:** Barbara Burtness, Jean P Bourhis, Jan B Vermorken, Kevin J Harrington, Ezra EW Cohen

**Affiliations:** Yale University School of Medicine, 333 Cedar Street, New Haven, CT 06520 USA; Centre Hospitalier Universitaire Vaudois, Lausanne, Switzerland; Antwerp University Hospital, Edegem, Belgium; Head and Neck Unit, The Royal Marsden Hospital, London, UK; Division of Radiotherapy and Imaging, Institute of Cancer Research, London, UK; University of California San Diego Moores Cancer Center, La Jolla, CA USA

**Keywords:** Adjuvant, Afatinib, Head and neck, Local advanced, Phase III, Unfavourable risk

## Abstract

**Background:**

Over 50% of patients with head and neck squamous cell carcinoma (HNSCC) present with locoregionally advanced disease. Those at intermediate-to-high risk of recurrence after definitive therapy exhibit advanced disease based on tumour size or lymph node involvement, non-oropharynx primary sites, human papillomavirus (HPV)-negative oropharyngeal cancer, or HPV-positive oropharynx cancer with smoking history (>10-pack-years). Non-surgical approaches include concurrent chemoradiotherapy, induction chemotherapy followed by definitive radiotherapy or chemoradiotherapy, or radiotherapy alone. Following locoregional therapies (including surgical salvage of residual cervical nodes), no standard intervention exists. Overexpression of epidermal growth factor receptor (EGFR), an ErbB family member, is associated with poor prognosis in HNSCC. EGFR-targeted cetuximab is the only targeted therapy that impacts overall survival and is approved for HNSCC in the USA or Europe. However, resistance often occurs, and new approaches, such as targeting multiple ErbB family members, may be required. Afatinib, an irreversible ErbB family blocker, demonstrated antiproliferative activity in preclinical models and comparable clinical efficacy with cetuximab in a randomized phase II trial in recurrent or metastatic HNSCC. LUX-Head & Neck 2, a phase III study, will assess adjuvant afatinib versus placebo following chemoradiotherapy in primary unresected locoregionally advanced intermediate-to-high-risk HNSCC.

**Methods/design:**

Patients with primary unresected locoregionally advanced HNSCC, in good clinical condition with unfavourable risk of recurrence, and no evidence of disease after chemoradiotherapy will be randomized 2:1 to oral once-daily afatinib (40 mg starting dose) or placebo. As HPV status will not be determined for eligibility, unfavourable risk is defined as non-oropharynx primary site or oropharynx cancer in patients with a smoking history (>10 pack-years). Treatment will continue for 18 months or until recurrence or unacceptable adverse events occur. The primary endpoint measure is duration of disease-free survival; secondary endpoint measures are disease-free survival rate at 2 years, overall survival, health-related quality of life and safety.

**Discussion:**

Given the unmet need in the adjuvant treatment of intermediate-to-high-risk HNSCC patients, it is expected that LUX-Head & Neck 2 will provide new insights into treatment in this setting and might demonstrate the ability of afatinib to significantly improve disease-free survival, compared with placebo.

**Trial registration:**

ClinicalTrials.gov NCT01345669.

**Electronic supplementary material:**

The online version of this article (doi:10.1186/1745-6215-15-469) contains supplementary material, which is available to authorized users.

## Background

The most common type of malignant tumour of the head and neck is squamous cell carcinoma, with an estimated global incidence of approximately 600,000 cases per year [1]. Locoregionally advanced disease (stage III to IVb) accounts for more than 50% of patients diagnosed with this cancer; 40% of patients present with early-stage disease and 10% present with metastatic disease at diagnosis [2]. Five-year overall survival rates vary in patients with locoregionally advanced disease. Overall survival ranges from 10% to 75% depending on tumour stage, the site of the primary tumour, human papillomavirus (HPV) association and other known risk factors [3–7].

For oropharyngeal squamous cell carcinoma (OPSCC), high-risk patients with locoregionally advanced disease have been previously defined as patients with HPV-negative tumours who either have a ≤10 pack-year smoking history with T4 tumours (indicating invasion of the primary tumour into adjacent tissues and structures, based on the tumour-node-metastasis (TNM) Staging Classification for Head and Neck Cancers) [8] or have a >10 pack-year smoking history irrespective of primary tumour stage [9, 10]. Patients with HPV-positive OPSCC and a >10 pack-year smoking history also have a worse prognosis and are defined as being at an intermediate risk of death. This risk stratification model was confirmed in a 2012 study by Broglie *et al.*[11]. Human papillomavirus infection has emerged as an aetiological factor in around 20 to 25% of head and neck squamous cell carcinoma (HNSCC) [7, 12], with HPV being strongly prognostic for survival in OPSCC [9]. Human papillomavirus status also differs substantially with geographical location, with HPV prevalence in OPSCCs found to be higher in North America and Asia than in Europe [13]. However, this may be confounded by testing only for the HPV types that are most common in North America, and therefore other high-risk types of HPV may be more prevalent elsewhere in the world.

p16 is a tumour suppressor and cell-cycle regulator that is upregulated in HPV-positive OPSCC and often lost in HPV-negative OPSCC. In OPSCC, p16 expression is well established as a surrogate marker of HPV infection and is also associated with prognosis [14, 15]. Indeed, patients with p16-positive oropharyngeal cancer have significantly improved failure-free survival and overall survival versus patients with p16-negative oropharyngeal cancer (failure-free survival hazard ratio = 0.28, *P* < 0.001 and overall survival hazard ratio = 0.31, *P* = 0.002) [16]. Recent evidence also suggests that patients with p16-positive OPSCC have improved progression-free survival and overall survival compared with p16-positive patients with non-OPSCC, but patients with p16-negative OPSCC and non-OPSCC have similar outcomes [14].

Overall survival in patients with high-risk locoregionally advanced OPSCC is low; in the Radiation Therapy Oncology Group 0129 trial reported by Ang *et al.*[9], these patients demonstrated a 3-year overall survival rate of 46.2% following concurrent chemoradiotherapy, compared with 93% in a low-risk group and 70.8% in an intermediate-risk group. In a study reported by Broglie *et al.* in 2012 [11], similar figures were described: 3-year survival rates of 65% in a high-risk group, 88% in a low-risk group and 77% in an intermediate-risk group. In the Radiation Therapy Oncology Group 0129 study, patients with HPV-negative OPSCC had a significantly worse 3-year survival rate (57.1% versus 82.4%; *P* < 0.001), a worse 3-year progression-free survival rate (43.4% versus 73.7%; *P* < 0.001), significantly more locoregional relapses (35.1% versus 13.6%; *P* < 0.001) and significantly more second primary tumours (14.6% versus 5.9%; *P* = 0.02) versus patients with HPV-positive OPSCC. Therefore, because of the available data on the prognostic factors in OPSCC, both patients with p16-negative locoregionally advanced OPSCC and patients with non-OPSCC locoregionally advanced disease were considered to have high-risk locoregionally advanced HNSCC.

### Non-targeted treatment of locoregionally advanced HNSCC

Following diagnosis of primary locoregionally advanced HNSCC, standard treatment options are concurrent chemoradiotherapy, radiotherapy alone, induction chemotherapy followed by radiotherapy, or concurrent chemoradiotherapy [8], with cisplatin being the most common chemotherapy used in combination with radiotherapy [2]. In a meta-analysis by Pignon *et al.*[17], an absolute overall survival benefit of 6.5% at 5 years was associated with concomitant chemoradiotherapy; the benefit was only 1% for adjuvant chemotherapy and 2% for neoadjuvant chemotherapy in patients previously untreated for locoregionally advanced disease. Although there is a genuine improvement in overall survival following concurrent chemoradiotherapy, the overall survival benefit is still low. Furthermore, as mentioned previously, the 3-year overall survival observed for concurrent chemoradiotherapy in patients with untreated HPV-negative oropharyngeal cancer is only 57% and the risk of locoregional relapse is 35% [9].

The use of chemotherapy after locoregional treatment has been studied in only a limited number of randomized trials in HNSCC. Early studies examining the impact of chemotherapy in the maintenance setting demonstrated modest, but minimal, benefits, and these did not translate into a survival benefit overall [18–20]. A significant survival advantage was seen only in a subgroup of patients with N2 disease in a subset analysis of the Head and Neck Contracts Programme, suggesting that maintenance chemotherapy may only be appropriate for patients with advanced disease [18]. A meta-analysis of 23 chemotherapy trials evaluating the timing of chemotherapy (including 10 trials with maintenance treatment) suggested that cancer mortality was only influenced by both synchronous and maintenance chemotherapy [21]. However, most of the trials in this meta-analysis were small, and a direct comparison of maintenance versus no maintenance was not conducted. Toxicities in these combined modality treatments were also substantial and dose-limiting. Therefore, despite signals of some survival benefit, the use of cytotoxic chemotherapy after locoregional treatment has not gained acceptance within clinical practice. With the more recent advent of targeted therapies (in particular those that can be orally administered), re-examination of the question of continuing systemic therapy beyond the completion of chemoradiation is warranted.

#### Targeted treatment approaches

Epidermal growth factor receptor (EGFR; ErbB1) is expressed in over 90% of HNSCCs [22], with high levels of EGFR protein expression being associated with decreased survival, resistance to radiotherapy, locoregional treatment failure and high rates of distant metastases [23]. A 2011 study showed that patients with HNSCC and high EGFR protein expression display inferior 5-year overall survival rates, compared with patients with HNSCC and low EGFR protein expression (*P* = 0.029) [24].

Cetuximab, an EGFR-targeting monoclonal antibody, is the only targeted treatment approved in the USA, Japan and Europe for HNSCC, where it is approved in combination with radiotherapy for the treatment of locoregionally advanced disease [25, 26]. In the same regions, cetuximab is also approved for the first-line treatment of recurrent or metastatic HNSCC in combination with platinum-based chemotherapy. The US label also indicates cetuximab monotherapy for the second-line treatment of recurrent or metastatic HNSCC following failure of platinum-based chemotherapy [25, 26].

In patients with untreated locoregionally advanced HNSCC, cetuximab plus radiotherapy compared with radiotherapy alone showed a statistically significant improvement in the primary endpoint of locoregional control (median, 24.4 months versus 14.9 months; *P* = 0.005), as well as the secondary endpoints of overall survival (median, 49 months versus 29.3 months; *P* = 0.03) and progression-free survival (median, 17 months versus 12 months; *P* = 0.006) [27]. Cetuximab also impacts survival in the recurrent or metastatic setting. The proof-of-principle study conducted by the Eastern Cooperative Oncology Group (ECOG) compared cisplatin plus cetuximab versus cisplatin plus placebo in patients with measurable or evaluable recurrent or metastatic HNSCC; this demonstrated a significant improvement in objective response rate (26% versus 10%), but was underpowered for progression-free survival and overall survival [28]. A larger (*n* = 442) phase III study, in which cetuximab plus platinum/5-fluorouracil was compared with platinum/5-fluorouracil alone, did show an impact on survival, demonstrating an improvement from 7.4 months for the control arm to a median of 10.1 months for the cetuximab arm [29]. However, median overall survival still remains unsatisfactory in the recurrent or metastatic setting and there is an urgent need for further improvement.

Extensive cross-talk between all ErbB-dependent signalling pathways in HNSCC, as well as the numerous molecular and genetic aberrations present, contribute to the development of cetuximab resistance [30]. Acquired resistance to cetuximab has been linked to dysregulation of EGFR internalization or degradation, EGFR-dependent activation of human epidermal growth factor receptor 2 (HER2; ErbB2) and ErbB3, and increased signalling of alternative receptor tyrosine kinases, such as cMET [31]. To overcome treatment resistance, new investigational therapies target more than one member of the ErbB family of receptors simultaneously, reversibly or irreversibly [23]. Lapatinib, a dual reversible EGFR/HER2 small-molecule tyrosine kinase inhibitor, is currently being assessed in the maintenance setting versus placebo (both in combination with chemoradiotherapy) in a phase III study in patients with high-risk HNSCC who have undergone resection [32]. Results are expected in mid-2014. Another strategy is irreversible blockade of all members of the ErbB family, which might compromise all ErbB-mediated signalling pathways and cause sustained blockade of ErbB receptor dimers. This might lead to suppression of tumour growth and, it is expected, improved efficacy. Given the central role of the ErbB family in the tumourigenesis of HNSCC, this is an attractive target for treatment in the adjuvant setting. This may be especially useful when an aggressive multimodal treatment approach is needed to combat further tumour growth and prevent the development of metastatic disease.

#### Afatinib, an irreversible ErbB family blocker

Afatinib is an oral, irreversible ErbB family blocker that completely and irreversibly blocks signalling by EGFR, HER2 and ErbB4. It also blocks transphosphorylation of ErbB3 [33, 34]. Afatinib is approved in the major ICH (International Conference on Harmonisation of Technical Requirements for Registration of Pharmaceuticals for Human Use) regions of the USA, EU and Japan for the treatment of patients with non-small cell lung cancer harbouring distinct EGFR activating mutations [35–37]. Afatinib is also undergoing development as a therapy for several other ErbB-driven cancers. Approval was based on findings from the pivotal phase III LUX-Lung 3 study, which demonstrated a median progression-free survival of 11.1 months in patients treated with afatinib versus 6.9 months in patients treated with chemotherapy (*P* < 0.001) in the first-line EGFR mutation-positive setting [38]. In more recent analyses, an overall survival benefit with afatinib versus chemotherapy was also observed in patients with non-small cell lung cancer harbouring the EGFR Del19 mutation [39].

Preclinical activity of afatinib in HNSCC has been observed in human HNSCC FaDu mouse models [40], whilst *in-vitro* combination experiments demonstrated additive activity of afatinib when combined with standard chemotherapy [41]. Furthermore, proof of concept for afatinib in HNSCC has been demonstrated in a phase II crossover study of afatinib versus cetuximab, in which comparable activity of both agents was observed in patients with recurrent or metastatic HNSCC for whom platinum-based chemotherapy had failed. Objective response rates in stage 1 of the study were 8.1% in the afatinib group and 9.7% in the cetuximab group (independent central review) [42]. In stage 2 of the study, after crossover had occurred, the disease control rate (including complete response, partial response and stable disease) was 33% in afatinib-treated patients versus 19% in cetuximab-treated patients [42]. Furthermore, in stage 2 of the study, the progression-free survival time was 9.3 weeks for afatinib-treated patients (following cetuximab in stage 1 of the study) and 5.7 weeks in cetuximab-treated patients (following afatinib in stage 1 of the study). Although stage 2 of the study was not powered to detect a significant difference in progression-free survival between treatment groups, these data suggest that sequential therapy with EGFR- or ErbB family-targeted agents might have clinical benefit in patients with recurrent or metastatic HNSCC.

Gastrointestinal and dermatological side effects are common in patients receiving EGFR tyrosine kinase inhibitors, and have a large impact on patients’ quality of life. Afatinib has a manageable tolerability profile in clinical trials, with toxicity dominated particularly by gastrointestinal and dermatological side effects. Recent pooled data analyses suggest that adverse events associated with afatinib can be effectively managed in patients with solid tumours [43, 44].

The dosage for afatinib was initially established during monotherapy trials, with a maximum tolerated daily dose of either 40 or 50 mg [45, 46]. LUX-Lung 2 evaluated 50 mg/day afatinib in patients with EGFR mutation-positive non-small cell lung cancer who had received ≤1 prior chemotherapy regimen. This dose was reduced to 40 mg/day following a protocol amendment to improve afatinib’s tolerability profile [47]. No difference in activity between these doses was observed in patients in LUX-Lung 2 and thus 40 mg/day afatinib was selected as the starting dose in the LUX-Lung 3 and 6 studies [38, 48, 49]. In the HNSCC proof-of-concept phase II trial, the starting dose of 50 mg/day afatinib was not as well tolerated as 40 mg/day afatinib. In stage 1 of this trial, 18 of 61 (29.5%) afatinib-treated patients had their dose reduced from 50 to 40 mg/day in response to an adverse event, with the most frequently reported adverse events that led to dose reductions being diarrhoea and rash. In stage 2 of the trial, 11 of 36 (30.6%) afatinib-treated patients experienced dose reductions to 40 mg/day following an adverse event; diarrhoea and rash were again the most common reason for dose reduction. Given the projected long duration of therapy in a group of patients who are likely to have low-grade persisting toxicities of chemoradiotherapy, an initial dose of 40 mg/day afatinib is utilized in LUX-Head & Neck 2, with individual dose modifications.

The LUX-Head & Neck 2 trial (NCT01345669) investigates the efficacy and tolerability of afatinib compared with placebo when given as adjuvant therapy after chemoradiotherapy, in patients with primary unresected HNSCC prior to chemoradiotherapy and no evidence of disease (with or without salvage tumour resection or neck dissection) post-chemoradiotherapy. The hypothesis being tested is that prolonged, irreversible, small-molecule inhibitor-mediated ErbB family blockade will improve disease-free survival outcomes. The study aims to select patients with unfavourable prognosis according to disease stage, tumour site and smoking status or history.

## Methods/Design

### Objectives

The primary objective of this study is to evaluate the superiority of afatinib as adjuvant therapy versus placebo in terms of disease-free survival (primary endpoint) following chemoradiotherapy in patients with primary unresected locoregionally advanced (stage III, IVa or IVb) HNSCC. Secondary endpoints include the disease-free survival rate at 2 years, overall survival rate, health-related quality of life and safety. Voluntary biomarker assessments were also conducted.

### Study design and treatments

**Figure 1:**
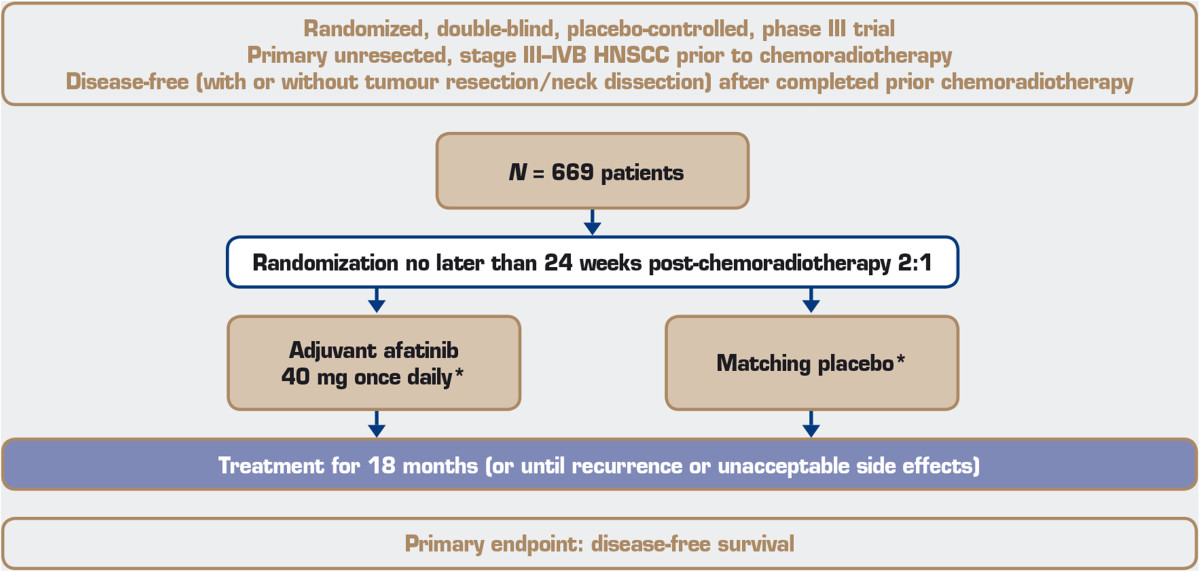
**Trial design.**

The LUX-Head & Neck 2 trial is a randomized, multicentre, double-blind, placebo-controlled, phase III trial. Patients will undergo magnetic resonance imaging, positron emission tomography or computed tomography approximately 8 to 12 weeks after completion of chemoradiotherapy and, if there is no evidence of disease, patients will be randomized no later than 24 weeks post-chemoradiotherapy to either afatinib or placebo in the ratio 2:1. Randomization will be conducted centrally with a validated random number-generating system at Boehringer Ingelheim, verified by a trial-independent statistician, and implemented via an interactive internet and voice-response system. Access to the randomization code will be supervised by the clinical trial support group; those directly involved in the conduct and analysis of the trial will not have access to the randomization schedule prior to database lock. Stratification will be based on patients’ nodal status (N0 to N2a versus N2b to N3, based on the TNM Staging Classification for Head and Neck Cancers) [8] and ECOG performance status (0 versus 1) at randomization. Eligible patients will receive, as adjuvant therapy, either continuous oral once-daily afatinib at a starting dose of 40 mg or placebo, which will be administered for 18 months or until disease recurrence or unacceptable adverse events occur. The dose of afatinib will be escalated to 50 mg in patients with no or minimal drug-related adverse events or will be reduced to 40 mg (if first escalated to 50 mg), 30 mg or 20 mg in patients experiencing drug-related adverse events (Figure 1).

This trial is expected to randomize 669 patients to treatment worldwide (446 in the afatinib arm and 223 in the placebo arm) and will be carried out in compliance with the protocol, and in accordance with the principles laid down in the Declaration of Helsinki, the International Conference on Harmonisation Guideline for Good Clinical Practice and applicable regional-specific regulatory requirements. The study protocol has been reviewed by an independent ethics committees (please see Additional file 1 for details of all approving committees), according to national and international regulations, and written informed consent will be obtained from each patient before any study-specific screening assessments are performed. An independent data monitoring committee will oversee the trial and will advise on the further conduct of the trial based on ongoing evaluation of efficacy and safety data.

### Patients

Eligible patients must be ≥18 years of age, have an ECOG performance status of 0 or 1 and have histologically or cytologically confirmed locoregionally advanced HNSCC at the time of randomization (stage III, IVa or IVb squamous cell carcinoma of the oral cavity, oropharynx or hypopharynx, or stage IVa or IVb squamous cell carcinoma of the larynx). Patients are required to have unresected disease prior to chemoradiotherapy and an unfavourable risk of recurrence. Patients can have unresected disease for the following reasons: (1) they may have technically unresectable disease owing to tumour fixation, or invasion to either base of the skull, cervical vertebrae, nasopharynx or fixed lymph nodes; (2) there may be low surgical curability (T3 or T4, N2 or N3 excluding T1N2, based on the TNM Staging Classification for Head and Neck Cancers) [8]; or (3) patients may be treated for organ preservation. As HPV status will not be determined for eligibility in this study, unfavourable risk is defined as non-oropharynx primary site or oropharynx cancer in heavy smokers (>10 pack-years). Patients must also have completed prior definitive platinum-based chemoradiotherapy by no longer than 24 weeks prior to randomization (at least two cycles of cisplatin at a minimum cumulative dose of 200 mg/m^2^ or carboplatin at a minimum cumulative area under the concentration-time curve of 9; radiotherapy of minimum 66 Gy in 33 fractions (or its radiobiological equivalent)), and at randomization have chemoradiotherapy-induced adverse events classified as less than or equal to grade 2 using the Common Terminology Criteria for Adverse Events. Patients are required to have no evidence of disease after chemoradiotherapy (that is, no residual tumour after chemoradiotherapy, or no residual tumour after chemoradiotherapy followed by R0 tumour resection, or no evidence of nodal disease after chemoradiotherapy followed by neck dissection). Patients must also have adequate bone marrow, liver and kidney function. The main exclusion criteria are prior treatment with EGFR-targeted small molecules, EGFR-targeted antibodies or any investigational agents for HNSCC, patients with a smoking history of ≤10 pack-years and with primary tumour site of base of tongue or tonsil, and primary cancer of nasopharynx, sinuses or salivary glands.

### Efficacy assessments

Disease-free survival, the primary endpoint measure, is defined as the time from randomization until documented tumour recurrence or second primary tumour or death of any cause, whichever occurs first. The definition of recurrence or second primary tumour is the appearance of any new lesions without any evident benign aetiology, assessed by imaging of the head and neck and chest by the investigator and an independent central review. The primary analysis will be based on the investigator’s assessment. Tumour recurrence will be further classified into subtypes of local, regional or distant recurrence. Disease-free survival rate at 2 years, the key secondary endpoint, will be calculated in each group using the Kaplan-Meier method. Overall survival is defined as time from the date of randomization until death.

### Safety assessments

The incidence and intensity of adverse events will be evaluated according to the National Cancer Institute Common Terminology Criteria for Adverse Events version 3.0. All adverse events, serious and non-serious, occurring during the course of the trial (that is, from randomization until 28 days after end of treatment), regardless of relatedness to study medication, will be collected, documented and reported by the investigator. Serious and non-serious adverse events occurring later than 28 days after the last administration of study medication will only be reported if they are considered relevant by the investigator. All adverse events, including those persisting after the end of the study medication, must be followed up until they have resolved or have been sufficiently characterized. Safety endpoints are the overall incidence and intensity of adverse events, gastrointestinal events (vomiting, nausea and diarrhoea), skin reactions (rash, acne) and change from baseline in all laboratory tests.

### Health-related quality of life assessment

Health-related quality of life will be assessed using the following patient-reported outcome measures: European Organisation for Research and Treatment of Cancer Quality of Life Questionnaire (QLQ-C30); European Organisation for Research and Treatment of Cancer Quality of Life Head and Neck cancer module (QLQ-H&N35); and EQ-5D health status questionnaire. Questionnaires will be completed at randomization and at each tumour assessment time point until disease recurrence. Questionnaires will be filled in by the patients at the clinic before any clinical assessment and treatment, and before provision of any new information about their disease status.

### Biomarker assessment

Participation in the biomarker analysis is voluntary and is not a prerequisite for participation in the trial. Separate informed consent must be provided by patients for this part of the study, in accordance with local ethical and regulatory requirements. Biomarker analyses will be performed using archival tumour tissue; fresh tumour biopsies are not possible as there is no residual disease in participating patients. Exploratory biomarkers to be assessed are p16 status, ErbB ligands, ErbB receptor expression, EGFR mutational or activation status and ErbB downstream signalling markers.

### Statistical analyses

This trial is powered to detect a prolonged median disease-free survival of 6.2 months with afatinib over an assumed disease-free survival of 15.8 months in the placebo arm [8]. A total of 669 patients randomized 2:1 to afatinib and placebo is required to detect a difference in disease-free survival (with a hazard ratio of 0.72) at a power of 90%, with a one-sided type-I error of α = 0.025.

Disease-free survival will be analyzed using the stratified log-rank test with nodal status (N0 to N2a versus N2b to N3) and ECOG performance status (0 versus 1) being the stratification factors. The Kaplan-Meier method will be used to estimate the 25th percentile, median and 75th percentile disease-free survival for each treatment group and the stratified Cox proportional hazards model will be used to derive the hazard ratio between the two treatment groups. The difference in the disease-free survival rate at 2 years between afatinib and placebo (*d*_KM_) will be calculated as the difference between the two Kaplan-Meier estimates. Overall survival will be analyzed using the stratified log-rank test, the Kaplan-Meier method and the stratified Cox proportional hazards model, in the same manner as for disease-free survival.

## Discussion

A detailed account of the LUX-Head & Neck 2 trial has been provided here to increase awareness of this study and provide a detailed rationale for assessment of afatinib in the adjuvant setting. It is important that patients with intermediate-to-high-risk primary unresected locoregionally advanced HNSCC are informed of the risk of recurrence or distant metastases following initial treatment. With the limited treatment options following chemoradiotherapy, sharing information on clinical trials of novel treatment options with patients is critical to provide them with the opportunity to participate in these studies.

The LUX-Head & Neck 2 trial will demonstrate whether afatinib reduces the risk of recurrent or metastatic disease following adjuvant treatment in patients with intermediate-to-high-risk primary unresected HNSCC, and will provide further knowledge on the treatment of this patient population. This is one of two phase III studies of afatinib in HNSCC; LUX-Head & Neck 1 (NCT01345682) is assessing afatinib versus methotrexate in patients with recurrent or metastatic HNSCC who have progressed with platinum-based therapy [50]. The primary endpoint for this trial has been completed and results from the study were presented at the European Society for Medical Oncology 2014 congress [51].

Novel activity of afatinib has been shown in preclinical models of head and neck cancer [40, 41] and comparable activity has been seen versus cetuximab in the proof-of-concept phase II trial [42]. Given these data and the clinical efficacy of afatinib in non-small cell lung cancer [38], similar data may be reflected here in the phase III LUX-Head & Neck 2 trial.

## Trial status

**Figure 2:**
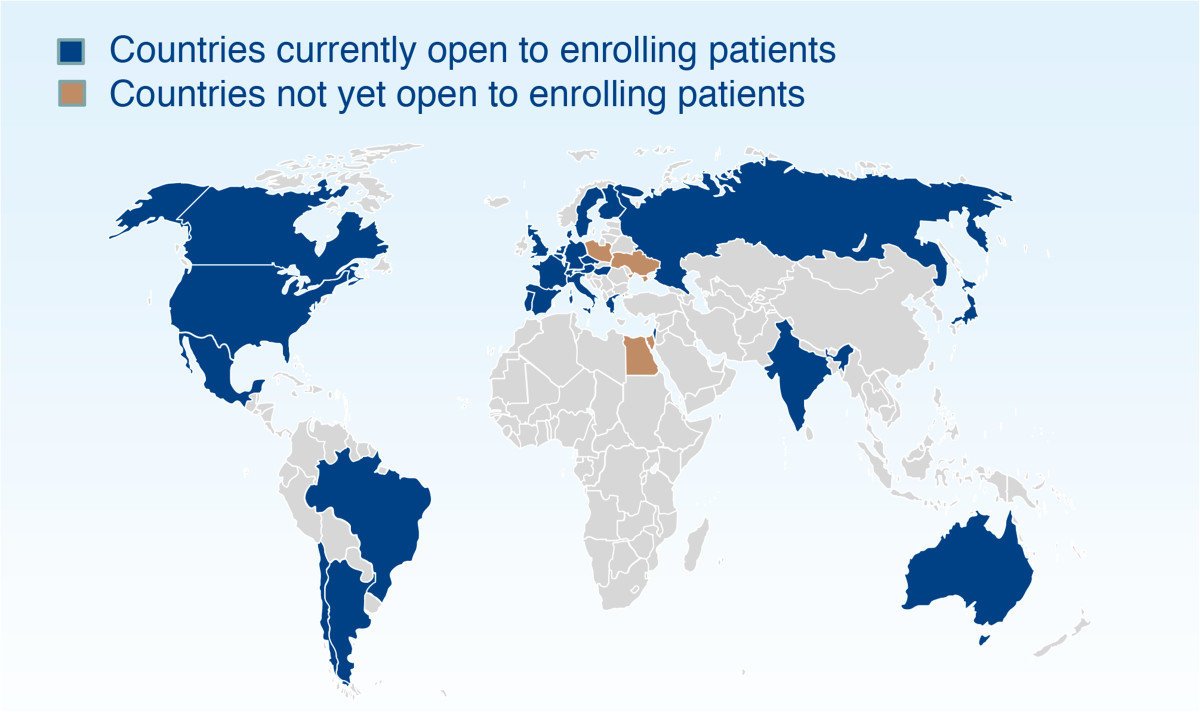
**Trial countries.**

The trial was initiated in October 2011 and is currently recruiting patients in Argentina, Australia, Austria, Belgium, Brazil, Canada, Chile, the Czech Republic, Denmark, Finland, France, Germany, Greece, Hungary, India, Israel, Italy, Japan, Mexico, Portugal, Russia, Spain, Sweden, Switzerland, the Netherlands, the UK and the USA (Figure 2). Other participating countries are Egypt, Poland and Ukraine.

## Electronic supplementary material

Additional file 1: List of approving institutional review boards and independent ethics committees.(DOCX 22 KB)

Below are the links to the authors’ original submitted files for images.

Authors’ original file for figure 1

Authors’ original file for figure 2

Authors’ original file for figure 3

Authors’ original file for figure 4

## Abbreviations

ECOG: Eastern Cooperative Oncology Group
EGFR: epidermal growth factor receptor
HER2: human epidermal growth factor receptor 2
HNSCC: head and neck squamous cell carcinoma
HPV: human papillomavirus
ICH: International Conference on Harmonisation of Technical Requirements for Registration of Pharmaceuticals for Human Use
OPSCC: oropharyngeal squamous cell carcinoma
TNM: tumour-node-metastasis.
