# Supplementary material for: Afatinib versus placebo as adjuvant therapy after chemoradiation in a double-blind, phase III study (LUX-Head & Neck 2) in patients with primary unresected, clinically intermediate-to-high-risk head and neck cancer: study protocol for a randomized controlled trial
Source: Trials. 2014 Nov 29;15:469. doi: 10.1186/1745-6215-15-469 (PMC4289298; doi:10.1186/1745-6215-15-469)
Supplement: Supplementary file 1 — Additional file 1: List of approving institutional review boards and independent ethics committees.(DOCX 22 KB) [file 13063_2014_2360_MOESM1_ESM.docx]

**Additional File 1.** List of approving institutional review boards and independent ethics committees

Argentina: Universidad de Buenos Aires – Instituto de Oncología Angel Roffo; Comité Independiente de Etica para Ensayos en Farmacología Clínica; Comité de Docencia e Investigación del ISIS centro Especializado; Comité de Docencia e Investigación; Cte de Etica del CER Investigaciones Clinicas; Comité de Revisión de Actividades de Investigación; Comité institucional de Etica de la Investigación en la Salud Clinica Colombo; Comité de Etica en Investigación Clinica

Australia: Metro South Hospital and Health Service Human Research Ethics Committee Centres for Health Research (reference number: HREC/11/QPAH/471)

Austria: Ethics Committee of the Medical University of Vienna (reference numbers: 738/2011,

738/2012, 738/2013, 738/2014, 738/2015, 738/2016, 738/2017)

Belgium: Ethisch Comité UZ Antwerpen (reference number: 11/24/186)

Brazil: Comitê de Ética em Pesquisa da Faculdade de Medicina da Universidade de São Paulo (reference number: 518/11); Comitê de Ética em Pesquisa em Seres Humanos do Hospital Pró-Cardíaco Pronto Socorro Cardiológico (reference number: 498); Comitê de Ética em Pesquisa em Seres Humanos da Irmandade da Santa Casa de Misericórdia de Porto Alegre/ RS (reference number: 3704/12); Comitê de Ética em Pesquisa em Seres Humanos da Fundação Pio XII - Hospital do Câncer de Barretos/SP (reference number: 652/2012); Comitê de Ética em Pesquisa em Seres Humanos da Fundação Hospital Amaral Carvalho – SP (reference number: 131/11); Comitê de Ética em Pesquisa em Seres Humanos da Fundação Antônio Prudente - Hospital do Câncer - AC Camargo/SP (reference number: 1600/11); Comitê de Ética em Pesquisa em Seres Humanos da Universidade de Passo Fundo/RS (reference number: 184/2012)

Canada: Windsor Regional Hospital Research Ethics Board; University of British Columbia BC Cancer Agency Research Ethics Board; McGill Faculty of Medicine; Comité d’Ethique Centre Hospitalier de l’Universite de Montreal; Western University Health Science Research Board

Chile: Comité Etico Científico HGF SSVQ; Comitpe Etico Científico Clínica Reñaca

Czech Republic: Ethics Committee of Hospital Bulovka (reference number: 31.7.2012/484/EK-Z); Ethics Committee of University Hospital Olomouc and Medical Faculty of Palacky University in Olomouc (reference number: 171/12 MEK 24); Ethics Committee for Multi-Centric Clinical Trial of the University Hospital Motol (reference number: EK-316/13); Ethics Committee of the Masaryk Memorial Cancer Institute (reference number: 08/13)

Denmark: De Videnskabsetiske Komiteer for Region Hovedstaden (reference number: H-4-2013-011)

Egypt: National Cancer Institute-Cairo University Institutional Review Board (reference number: 20100014005.2); Research Ethics Committee Faculty of Medicine-Cairo University (reference number: N-26-2014)

Finland: Ethics committee of Hospital District of Southwest Finland (reference number: ETMK: 76/180/2011)

France: Comité de Protection des Personnes Nord-Ouest III CHU (reference number: 2011-30)

Germany: Ethik-Kommission an der Medizinischen Fakultät der Universität Leipzig; Ethikkommission der Medizinischen Hochschule Hannover; Ethik-Kommission der Medizinischen Fakultät der Universität Duisburg-Essen; Ethikkommission der Friedrich-Schiller-Universität Jena; Ethikkommission an der Medizinischen Fakultät der Universität Rostock; Ethikkommission der Medizinischen Fakultät der Ludwig-Maximilians-Universität München; Ethikkommission bei der Landesärztekammer Rheinland-Pfalz; Ethik-Kommission der Albert-Ludwigs-Universität; Ethikkommission bei der Landesärztekammer Baden-Württemberg; Geschäftsstelle der Ethikkommission der Universität Ulm

Greece: National Ethics Committee (reference numbers: 48670, 55666, 67456, 133, 349)

Hungary: Egészségügyi Tudományos Tanács Klinikai Farmakológiai Etikai Bizottság (CEC) (reference numbers: 45584-0/2013-EKL, 25487-0/2014-EKL)

India: Institutional Ethics committee, Max Super Speciality Hospital; Ethics committee, S P Medical College & A G Hospitals, Bikaner; National Health & Education Society’s Clinical Research & Ethics committee PD Hinduja National Hospital & Medical Research Centre; Tata Memorial Hospital Mumbai; Sparsh Hospital & Criticila Care Pvt Ltd.; Human Research Ethics Committee H.M. Patel Centre for Medical Care and Education, Gokal; Institutional Ethics Committee, Andhra Pradesh; Ethical Review Board, Meenakshi Mission Hospital and Research Centre; Medical Ethics Committee, Dr. Kamakshi Memorial Hospital Pvt. Ltd.; Institutional Ethics Committee, Poona Medical Research Foundation; Institutional Ethics committee, Delhi State Cancer Institute; Institutional Ethics Committee of Deenanath mangeshkar Hospital and Research Centre; Institutional Ethics Committee, Yashoda Hospital; Institutional Ethics Committee, Fortis Memorial hospital and research centre; Institutional Ethics Committee, M. P. Shah Cancer Hospital

Israel: Helsinki Committee, Rambam Health Care Campus (reference number: 0437-11); Helsinki Committee, Belinson Campus, Rabin Medical Center (reference number: 6643); Helsinki Committee, Tel Hashomer (reference number: 9371-12)

Italy: Comitato Etico Indipendente, Fondazione IRCCS, Istituto Nazionale dei Tumori (reference number: INT 57/11); Comitato Etico Aziendale della ASL 2 Savonese di Savona (reference number: 36/2011/CE2); Comitato Etico, Istituto Nazionale per lo Studio e la Cura dei Tumori, Fondazione Senatore Pascale (reference number: 34/11); Comitato Etico, A.O. Spedali Civili di Brescia (reference number: 844); Comitato Etico, Dell´Azienda Ospedaliera S. Paolo di Milano (reference number: 22520); Comitato Etico,Dell´Azienda Ospedaliera S. Croce e Carle di Cuneo (reference number: 93/11); Comitato Etico, AUSL di Viterbo (reference number: 428); Comitato Etico, Universita Cattolica del Sacro Cuore Policlinico (reference number: 1125/11); Comitato Etico Dell’ IRCCS Istituto Europeo di Oncologia di Milano (reference number: R677 IEO S720/412); Comitato Etico Provinciale Azienda Ospedaliera Arcispedale Santa Maria Nuova (reference number: 138/2012)

Japan: The IRB of Hokkaido University Hospital; The IRB of Jichi Medical University Hospital; The IRB of National Cancer Center Hospital East; The IRB of National Hospital Organization Tokyo Medical Center; The IRB of Shizuoka Cancer Center; The IRB of Aichi Cancer Center Hospital; The IRB of Kobe University Hospital; The IRB of National Hospital Organization Shikoku Cancer Center; The IRB of Tokai University Hospital; The IRB of Osaka Medical Center for Cancer and Cardiovascular Diseases; The IRB of Hyogo Cancer Center; The IRB of Japanese Foundation for Cancer Research; The IRB of Miyagi Cancer Center

Mexico: Instituto Nacional de Cancerología – Comité de Bioética; Instituto Nacional de Cancerología – Comité Científico

Netherlands: Medisch Ethische Toetsings Commissie (METC) Erasmus MC (reference number: MEC-2011-328)

Portugal: Ethics Committee for Clinical Research (CEIC) (reference numbers: 20130214, 20130235, 20130278, 20130451)

Russia: Local Ethics Committee, Non-State Medical Institution: Central Clinical Hospital (reference number: 157); Ministry of Healthcare of Omsk Region Government Healthcare, Institution of Omsk Region: Clinical Oncology Center (reference number: 14); Expert Council for Ethics in Biomedical Research within State Budget Educational Institution of Higher Professional Education: Bashkir State Medical University under the Federal Agency for Healthcare; Local Ethics Committee within Regional Budget Medical Institution: Kursk Regional Clinical Oncological Center (reference number: 4); Ethics Committee within N.N. Blokhin Russian Cancer Research Center; Local Ethics Committee within State Budget Medical Institution of the Stavropol Territory: Pyatigorsk Oncological Center (reference number: 30); Ethics Committee within Federal State Budgetary Institution: Russian Radiology and Surgical Technologies Research Center under the Ministry of Healthcare of the Russian Federation (reference number: 03-13); The Ethics Committee within State-Funded Healthcare Institution

Republican Clinical Oncology Center under the Ministry of Healthcare of the Republic of Bashkrtostan (reference number: 6/1); Ethics Committee within St.Petersburg State Budgetary Healthcare Institution: Oncology Center of Moskovskiy District

Spain: CEIC Hospital Universitari de la Vall d´Hebrón; CEIC Hospital Universitari de Bellvitge,

A/A Secretaria Administrativa del CEIC; CEIC Hospital de la Santa Creu i Sant Pau, Servicio de Farmacología Clínica; CEIC Hospital Clínic i Provincial de Barcelona, Agencia de Ensayos Clínicos – Servicio de Farmacia; CEIC de Navarra, Secretaría del CEIC, Pabellón de docencia, Recinto Hospital de Navarra; CEIC Autonómico de Ensayos Clinicos de Andalucía; CEIC Área 11 – Hospital Universitario 12 de Octubre, Unidad de Gestión de Ensayos Clínicos; Comité Etico de Investigación Clínica de Aragón (CEICA); Comité Ético Investigación Clínica de Galicia, División de Farmacia y Productos Sanitarios, Conselleria de Sanidade; Hospital Universitario Puerta de Hierro de Majadahonda/Hospital Quiron de Madrid, Secretaría Técnica del CEIC; CEIC Área de Salud de Ávila, Hospital “Ntra. Sra. Sonsoles” - Registro General; CEIC Autonómico de Ensayos, Clinicos de Andalucía; CEIC Hospital Universitari de Girona Doctor Josep Trueta, Secretaría Técnica del CEIC; CEIC Hospital General Universitario Gregorio Marañón; CEIC Hospital Universitario, Ramón y Cajal, A/A Secretaría del CEIC

Sweden: Regionala Etikprövningsnämnden I Stockholm (reference number: 2011/1962-31/2)

Switzerland: Ethikkommission beider Basel EKBB (reference number: 123/12); Kantonale Ethikkommission Bern KEK (reference number: 026/12)

United Kingdom: London – Chelsea Health Research Authority National Research Ethics Service (NRES) (reference number: 11/LO/0954)

United States: Office of Research & Sponsored Programs, Little Rock, AR (reference number: 134922); Institutional Review Board Fox Chase Cancer Center (reference number: 11-058); 1620 McElderry Street, Reed Hall, Suite B-130, Baltimore, MD (reference number: NA 0000.69444); Dartmouth Medical School (reference number: 2.3.3.7.3); Dana-Farber Cancer Institute Institutional Review Board (reference number: 12-028); University of Colorado Hospital Research Review Committee (reference number: 113-6812); University of California Los Angeles Office of Human Research Protection Programs (reference number: 12-000411); Committee on Research Involving Human Subjects (reference number: 299461-4; CORIHS# 2012-1701-F); UC Irvine Office of Research (reference number: HS# 2012-8879); Methodist Hospital Omaha; 4445 Lake Forest Drive, Suite 300, Cinncinnati, OH (reference number: 201200263); Institutional Review Board, San Antonio, TX (reference number: HSC20120101H [Grant # CTRC# 11-59]); Human Investigation Committee, New Haven, CT (reference number: 1208010720); Louisiana State University Health Science Center New Orleans Institutional Review Board (reference number: 7944); Albert Einstein College of Medicine of Yeshiva University (reference number: 12-07-245); Ingalls Memorial Hospital Institutional Review Board; Orlando Health/Orlando Regional Healthcare System IRB (reference number: 14.018.02); Schulman Associates Institutional Review Board, Inc. (reference number: 201401434)
