# Supplementary figures and images for: Afatinib versus placebo as adjuvant therapy after chemoradiation in a double-blind, phase III study (LUX-Head & Neck 2) in patients with primary unresected, clinically intermediate-to-high-risk head and neck cancer: study protocol for a randomized controlled trial
Source: Trials. 2014 Nov 29;15:469. doi: 10.1186/1745-6215-15-469 (PMC4289298; doi:10.1186/1745-6215-15-469)

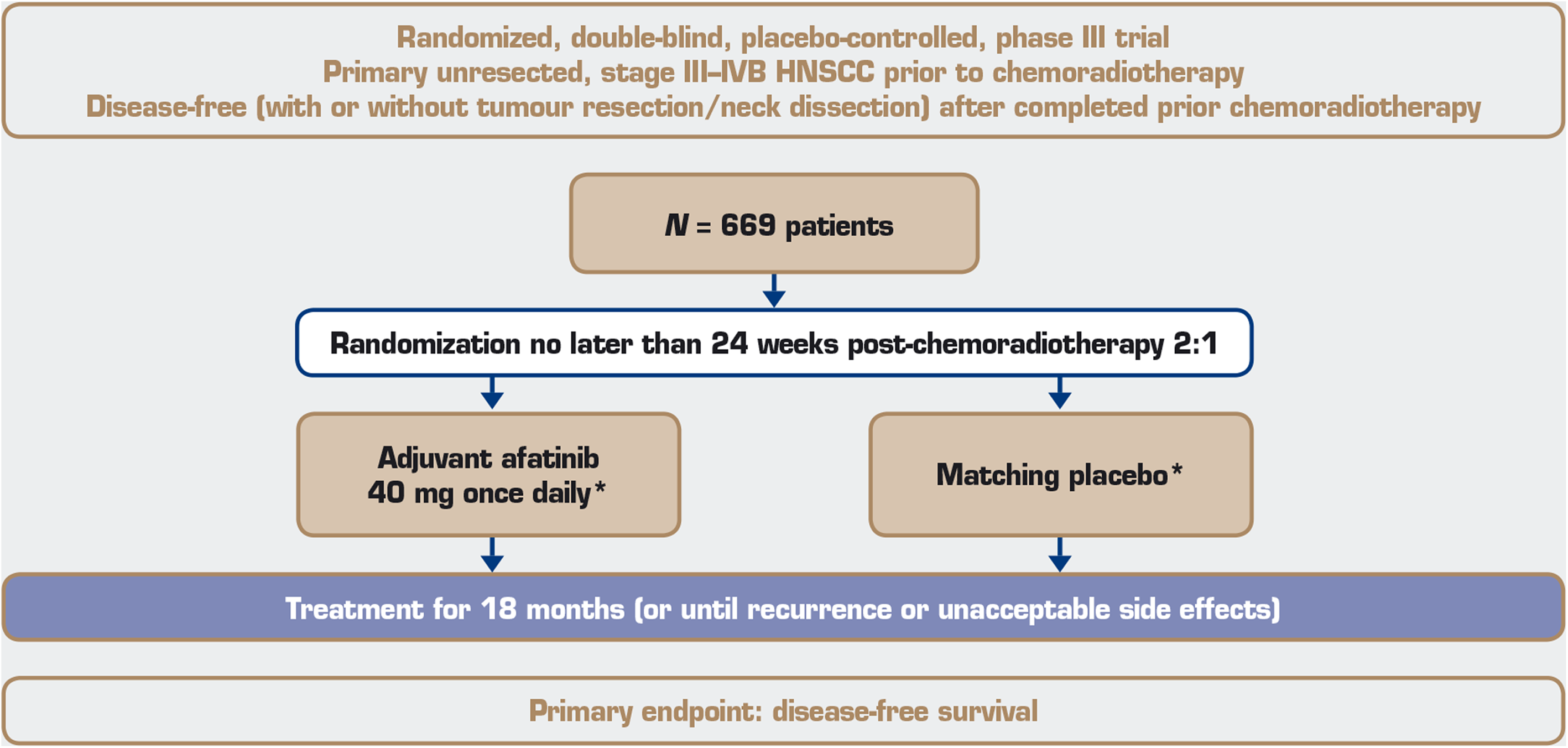

Supplement: Supplementary file 2 — Authors’ original file for figure 1 [file 13063_2014_2360_MOESM2_ESM.tiff]

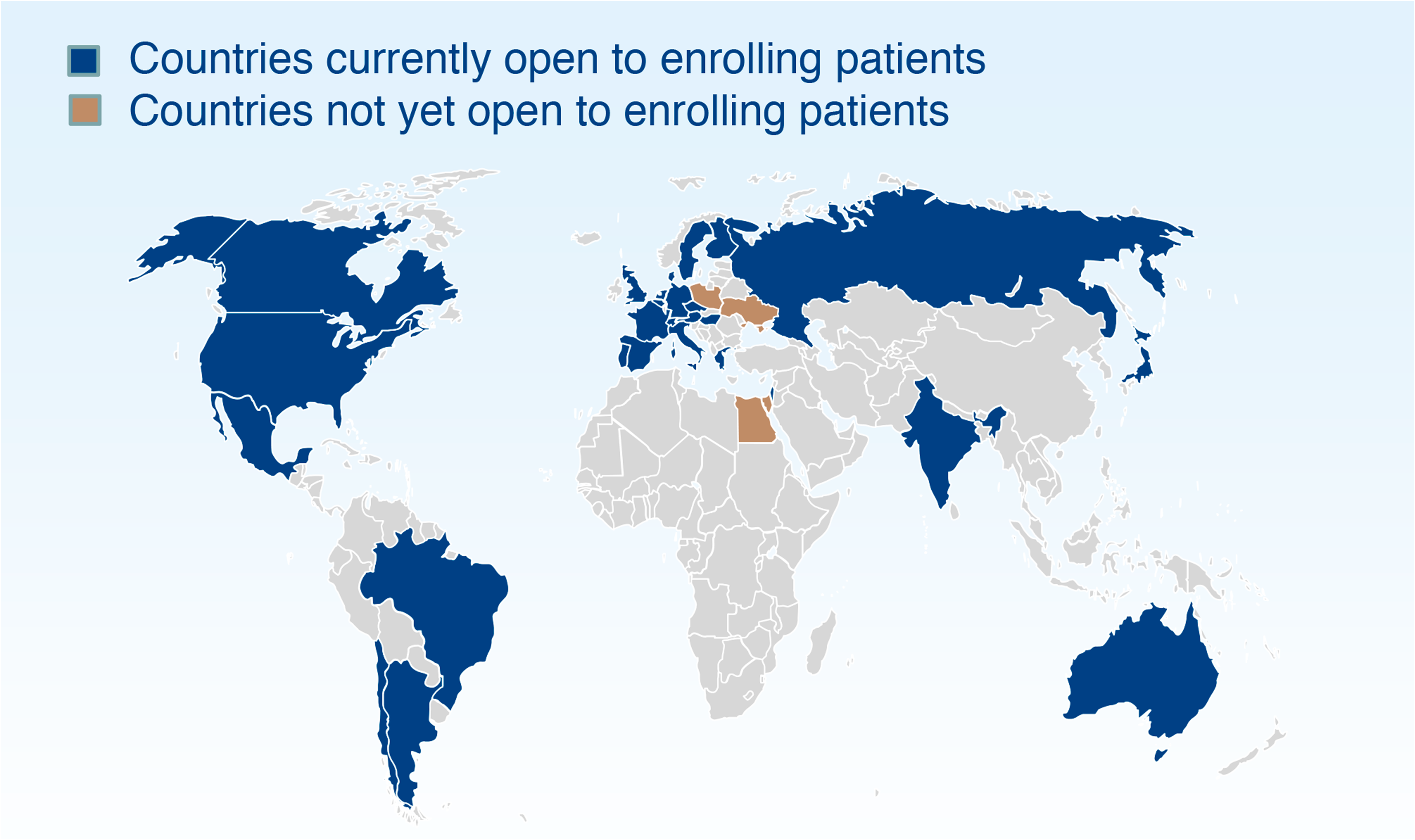

Supplement: Supplementary file 3 — Authors’ original file for figure 2 [file 13063_2014_2360_MOESM3_ESM.tiff]
